# Complex Presentation of a Dieulafoy Lesion in a Geriatric Patient with Multiple Comorbidities

**DOI:** 10.7759/cureus.47985

**Published:** 2023-10-30

**Authors:** Khadeejeh Alfroukh, Mohammed E Janajri, Aya E Mikkawi, Zinah Bairmani, Qais M Salah, Abdallatif M Dawoud, Majdeddin MohammedAli, Abdelwadod A Abuturki, Aref Al-Rajabi

**Affiliations:** 1 Internal Medicine, Al-Ahli Hospital, Hebron, PSE; 2 Internal Medicine, An-Najah National University, Nablus, PSE; 3 Pharmacology and Experimental Therapeutics, Thomas Jefferson University, Philadelphia, USA; 4 Internal Medicine, Al-Quds University, Jerusalem, PSE; 5 Medicine, An-Najah National University, Nablus, PSE; 6 Gastroenterology, Palestine Polytechnic University, Hebron, PSE

**Keywords:** intervention, comorbidity, elderly patient, gastrointestinal bleeding, dieulafoy lesion

## Abstract

Dieulafoy lesions (DL) consist of tortuous, thick-walled submucosal arteries that protrude through a small mucosal defect, often surrounded by otherwise normal mucosa. They are commonly located in the proximal stomach, particularly along the lesser curvature and near the esophagogastric junction, typically within 5 cm. However, they can also occur in various other regions of the GI tract, including the esophagus, duodenum, and colon. We present the case of a 76-year-old female with a complex medical history who arrived at the ED with hematemesis and melena. Her condition rapidly deteriorated; her blood pressure significantly dropped. Upon stabilization, upper endoscopy uncovered a 5-cm red lesion near the gastroesophageal junction, indicative of DL. Immediate intervention with clips was successful. Following the procedure, while the patient was in the ICU, she started to experience left-sided chest pain and diaphoresis, leading to the suspicion of acute coronary syndrome. Further investigations revealed non-ST-elevation myocardial infarction (NSTEMI). This case highlights the life-threatening nature of upper GI bleeding, especially in elderly patients with multiple comorbidities and extensive medication regimens. Timely diagnosis and intervention for DL are crucial, particularly in elderly patients with multiple health comorbidities. This underscores the significance of prompt medical attention and intervention in such complex scenarios.

## Introduction

A Dieulafoy lesion (DL) is a vascular abnormality characterized by large, tortuous submucosal arteries, which may rupture, leading to significant GI bleeding. Within the spectrum of acute GI bleeding causes, they represent a minor yet critical fraction, accounting for 1-2% of cases, but have a mortality rate between 9% and 13%, reaching up to 80% [1]. Diagnosis is often challenging due to the nature of these lesions; they commonly manifest as massive, intermittent arterial hemorrhages that obscure the active source. Additionally, their small size and lack of surrounding mucosal ulceration frequently necessitate multiple diagnostic and endoscopic interventions for identification [2]. The lesions may appear as either prominent, linear, or serpiginous submucosal arteries and sometimes can be identified incidentally [3].

Though advancements have been made in endoscopic therapeutic techniques, the etiology remains unclear, with associations with alcoholism and antiplatelet drug usage reported in the literature [1]. Given the diagnostic challenges and the potentially severe outcomes, these lesions hold significant interest for both gastroenterologists and internal medicine physicians [2]. DLs must be considered in the differential diagnosis of patients presenting with unclear etiology of GI bleeding, as rapid recognition and management are crucial to prevent life-threatening complications.

## Case presentation

We present the case of a 76-year-old female patient with a medical history including bronchial asthma, hypertension, and an ischemic stroke five years ago, resulting in more pronounced residual deficits in her left lower limb compared to her upper limb. She also has a history of heart failure with preserved ejection fraction (HFpEF) at 60% and pulmonary hypertension with a pulmonary artery pressure of 50. Furthermore, she recently experienced a deep vein thrombosis in her left leg.

The patient's current medication regimen includes regular inhalation therapies for asthma and consistent oral intake of rivaroxaban 20 mg once daily, aspirin 100 mg once daily, amlodipine 5 mg once daily, and enalapril 10 mg once daily. Additionally, she occasionally takes omeprazole 40 mg and ibuprofen, though not on a regular basis.

Upon presentation at the ED, the patient reported three episodes of coffee-ground hematemesis over the past three days, accompanied by three episodes of melena. The most recent hematemesis episode occurred on the same day as admission, involving significant blood loss. Palpitations, fatigue, and epigastric pain were also reported. Her initial vital signs showed a blood pressure of 120/70 mmHg and a pulse rate of 110 bpm. She was afebrile, and her oxygen saturation on room air was 94%.

Physical examination revealed orthostatic hypotension, pallor, and tachycardia. Muscle strength in her left upper and lower limbs was diminished to ⅗. Mild swelling in the left leg and epigastric tenderness were noted.

Upon admission, the patient received two wide-bore cannulas and intravenous fluids. A blood sample was promptly collected for an urgent CBC test and crossmatch in preparation for potential red blood cell transfusion based on CBC results.

The urgent CBC revealed a low hemoglobin level of 8.5 g/dL (refer to Table 1), which had dropped from 13 g/dL one month prior. Other laboratory results indicated a normal coagulation profile. Following the administration of approximately 1 liter of IV fluid, the patient's blood pressure stabilized at 125/80 mmHg, with a mean arterial pressure of 73 mmHg. However, tachycardia persisted at a rate of 105 bpm. Subsequently, the patient was expeditiously transferred to the ICU.

**Table 1:** Urgent blood tests carried out upon patient admission

Lab test	Result	Reference range
Hemoglobin	8.5	12-16 g/dL
Mean corpuscular volume	90	80-100 fL
Platelet count	255	150000-400000/mm 3
White blood cell count	12	5000-1000/mm 3

In the ICU, continuous monitoring encompassed the patient's airway, clinical status, vital signs, cardiac rhythm, urine output, and supplemental oxygen. A nasogastric tube was inserted, and isotonic crystalline infusions were administered. A unit of packed red blood cells (400 ml) was transfused over a three-hour period, aligning with CBC results from the ED, indicating a hemoglobin level of 8.5. Given the patient's history of heart failure and the elevated transfusion threshold set at 10 g/dL, this blood transfusion was deemed medically necessary due to the patient's symptomatic presentation. Additionally, a pantoprazole infusion of 8 mg/hour was initiated. Liver and kidney function tests yielded results within normal limits, and an abdominal ultrasound revealed no evidence of organomegaly.

**Figure 1:**
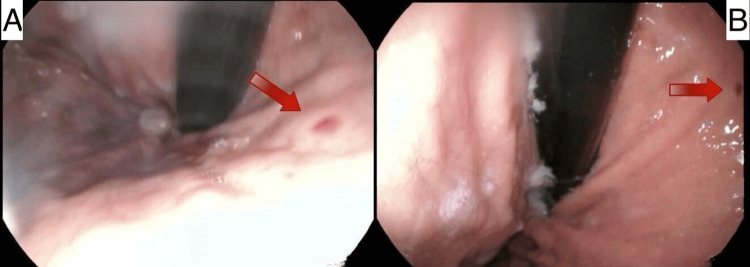
Two endoscopic views of the DL (red arrows)

**Figure 2:**
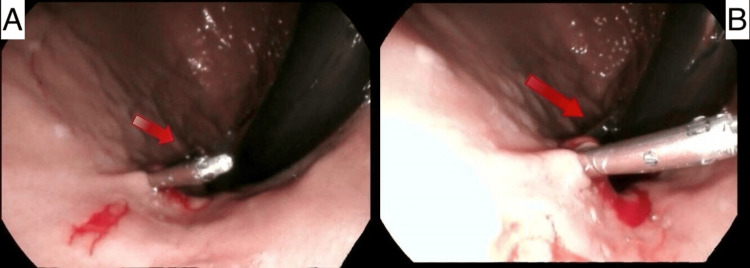
Two endoscopic views of the DL to which two hemostatic clips have been applied (red arrows)

Following hemodynamic stabilization, the patient underwent esophagogastroduodenoscopy (EGD), which revealed a 5 cm red lesion near the gastroesophageal junction consistent with Dieulafoy syndrome (refer to Figure 1A-2B). Two clips were inserted during the procedure (refer to Figure 2A-2B). The patient remained in the ICU for 48 hours for close monitoring; vital signs and hemoglobin levels remained stable during that time (refer to Table 2). The patient complained of chest pain and diaphoresis, and she received a diagnosis of on-ST-elevation myocardial infarction (NSTEMI) based on troponin levels and EKG findings indicating ST depression in inferolateral leads. Despite a comprehensive discussion recommending further evaluation and management, the patient chose to be discharged against medical recommendations.

**Table 2:** Laboratory test results during ICU stay

Lab test	Result	Reference range
Hemoglobin	10.65	12-16 g/dl
Mean corpuscular volume	85	82-92 fL
White blood cell	8.7	5000-1000/mm 3
Platelet	231	150000-400000/mm 3
First reading troponin	37	Less than 30 ng/l
Second reading troponin	338.9	Less than 30 ng/l
Third reading troponin	709	Less than 30 ng/l

## Discussion

A DL is a potentially life-threatening cause of GI bleeding, characterized by abnormally enlarged submucosal blood vessels that tend to bleed without any associated ulceration or erosion [4]. Notably, they are an underdiagnosed and rare condition, with diagnostic challenges rooted in their sporadic nature of bleeding. These lesions can remain asymptomatic, surrounded by unremarkable mucosa, only to present upon rupture [5].

In the context of our patient, early and accurate identification of DL became critical, given the significant risks associated with the condition. The presence of a DL in our patient meant that they were susceptible to sudden and massive bleeding, which could have been catastrophic without prompt endoscopic intervention. Had this lesion not been diagnosed and treated specifically, our patient might have been at risk for more severe outcomes, emphasizing the importance of actively searching for these lesions during endoscopic evaluations, especially in patients with a high comorbidity index [6].

DLs have a predominant gastric distribution, mostly localized within the stomach at the lesser curvature, particularly within a 6 cm range of the gastroesophageal junction [4]. This localization correlates with the direct arterial supply from the branches of the left gastric artery. However, these lesions are not limited to the stomach; they have been identified in various extra-gastric locations like the duodenum, colon, jejunum, esophagus, surgical anastomosis sites [4], and even the bronchi, especially manifesting as recurrent hemoptysis in chronic smokers [1]. Our patient's lesion location near the gastroesophageal junction is consistent with prevalent sites reported in the literature [4].

Regarding diagnosis, while regular EGD is often adequate for identifying DLs, up to 33% require multiple endoscopies before accurate identification [5]. Other methods, including endoscopic ultrasound, capsule enteroscopy, and angiography, should be considered, especially when DL is suspected and the lesions remain elusive [2].

DLs have shown a gender predisposition, with men being more likely to develop these lesions compared to women [4]. It's also vital to understand the significance of comorbidities and medications in the context of DL. There are established associations with conditions like cardiovascular disease, chronic kidney disease, hypertension, peptic ulcer disease, and diabetes mellitus [4]. Furthermore, the relationship between DL and the chronic use of certain drugs, especially NSAIDs and anticoagulants, cannot be understated [4]. Given our patient's medication history, it becomes essential to elucidate the potential effects of NSAIDs and anticoagulants on DL. These drugs may exacerbate the bleeding risk, compounding the patient's overall prognosis.

In terms of etiology, DL's origin continues to be a matter of debate. Prevailing theories lean toward considering DL as a congenital anomaly. It is thought that the blood vessel, instead of narrowing as it approaches the mucosa, retains its caliber, resulting in an abnormally large vessel prone to bleeding [7]. Over time, this vessel can erode the mucosa, hiding in plain sight until it eventually ruptures [5].

The choice of treatment for DL has evolved over the years, with endoscopic methods now being the preferred approach over surgical and radiological interventions. This shift can be credited with the marked improvement in mortality rates related to DL, which have dropped from 80% to 9-13% in recent decades [1]. If a single endoscopic method proves insufficient due to the lesion's pulsatile nature, combined therapies, like local epinephrine and endoclips, are recommended, as monotherapies pose a significantly higher risk of rebleeding [1]. However, if endoscopic techniques fail, angiographic embolization is the next logical step [2]. Our patient's favorable outcome, without further episodes of melena post-therapy, underscores the efficacy of the chosen treatment approach.

Lastly, the patient's NSTEMI diagnosis, potentially resulting from hypovolemia-induced myocardial ischemia, adds another dimension of complexity to management. While a direct association between DL and myocardial infarction remains understudied, it is prudent for clinicians to be aware of such correlations, especially in elderly patients with cardiovascular comorbidities.

## Conclusions

This case report highlights the diagnostic and therapeutic challenges DLs pose, particularly in complex clinical scenarios involving elderly patients with multiple comorbidities. Timely recognition and intervention are critical to address life-threatening GI bleeding. This case underscores the importance of considering DLs as potential sources of acute GI bleeding, even in the presence of complicating factors, and emphasizes the significance of further research to deepen our understanding of this condition's etiology and optimal management strategies.
